# Relative age effect in the 2022–2023 World Fencing Championships

**DOI:** 10.3389/fspor.2025.1588316

**Published:** 2025-07-02

**Authors:** Fabiana Bonito, Joana Viães, Júlia Teles, Luis A. Flores, Xavier Iglesias, Maria Isabel Fragoso

**Affiliations:** ^1^ Interdisciplinary Center for Human Performance, Faculty of Human Motricity, University of Lisbon, Dafundo, Portugal; ^2^ Faculty of Human Motricity, University of Lisbon, Dafundo, Portugal; ^3^ Faculty of Physical Culture Sciences, Autonomous University of Chihuahua, Chihuahua, Mexico; ^4^ National Institute of Physical Education of Catalonia, University of Barcelona, Barcelona, Spain

**Keywords:** relative age effect, fencing, talent identification, athlete development, youth sports

## Abstract

**Introduction:**

This study aimed to investigate the presence of relative age effect (RAE) in the World Fencing Championships of the 2022–2023 season across three age categories.

**Methods:**

Data from the participants of the World Fencing Championships were collected from the International Fencing Federation, resulting in a total of 2,791 participants distributed according to the age categories: 713 cadets, 1,048 juniors, and 1,030 senior athletes. The data collected included the athletes’ birthdate, birth quartile, sex, weapon, age category, country, continental area, and world championship result. An athlete’s relative classification was computed using their competition classification and the total number of participants in the event. The chi-square goodness-of-fit test was performed to assess the presence of RAE, examining differences in birth quarter distribution across the total sample, for each sex, and for the 18 events. Follow-up analyses included standardized residuals, Cramér's *V* effect size, and odds ratios. In events where RAE was detected, the Kruskal–Wallis and Quade's non-parametric ANCOVA tests were used to compare athletes’ relative classification across birth quarters.

**Results:**

RAE was present, in the overall sample [*χ*^2^(3) = 16.142, *p* < 0.001, *V* = 0.044], according to sex [female: *χ*^2^(3) = 10.349, *p* = 0.016, *V* = 0.053; male: *χ*^2^(3) = 7.987, *p* = 0.046, *V* = 0.041], and was inconclusive when focusing on each event.

**Discussion:**

The complexity of results in individual sports and the lack of research in fencing makes it difficult to understand the relevance of RAE in this sport. Despite the lack and inconsistency of results in fencing, coaches should be aware of this effect.

## 1 Introduction

Sport is often organized into age groups based on cutoff dates, which, in the case of fencing, start on 1 January (1). These cutoff dates place individuals who may differ in age by 1 year or more into the same category (2), leading to the relative age effect (RAE). In fencing, the age gap can be up to 2 years in the cadet category and 3 years in the junior category. The RAE refers to age-related differences between individuals born in the same calendar year, where those born earlier tend to have an advantage over those born later (2–4). The advantages to older individuals include their physical growth, maturation, or experience (4).

The RAE was first reported by Barnsley and Thompson (5) in ice hockey players. Three main theories have been proposed to explain the RAE (6): (1) the “Matthew effect” suggests that parents are more likely to enroll their older children in sports first, giving them an early advantage in training and experience that compounds over time; (2) the “Pygmalion effect” refers to the expectations coaches place on more physically mature players, leading to preferential selection and increased opportunities; and lastly, (3) the “Galatea effect” occurs when expectations placed on a player are internalized, influencing them to perform in line with those expectations (6). Supporting these theories is the maturation hypothesis, which states that older children are more physically and cognitively mature (7).

The prevalence of RAE depends on the sport, competitive level (e.g., recreational to elite level), athlete's age, sex, and physical characteristics (8). It is more common at elite levels (9, 10), in highly competitive environments where many athletes compete for limited opportunities (11), in younger age categories (12, 13), and in male sports (10, 14, 15). RAE is a well-documented phenomenon in team sports, particularly in popular and physically demanding disciplines such as football, basketball, and ice hockey (3, 10, 12, 16, 17). In contrast, its presence in individual sports varies depending on the sport's physical and competitive demands. For example, it is generally absent in low-contact, less physically demanding sports like shooting (18), but more prevalent in physically demanding disciplines such as skiing (7), and certain track and field events, where RAE increases with performance level (8, 15). In addition, some sports like swimming show event-specific RAE patterns (14), while others, such as gymnastics and figure skating, tend to exhibit an inverse RAE (19).

In individual opposition and combat sports like fencing (20–22), RAE was observed among the top 100 female tennis players (23) as well as across different weight classes and age groups in judo (13). Despite being a combat sport, fencing is one of the few sports in this category that does not have weight classes. Sports like wrestling, judo, or taekwondo have been the focus of previous research on RAE (24). Although some researchers argue that weight classes can be a moderator against RAE (25–28), others still observed it (21, 24, 29). Adding to the inconsistency of findings in combat sports (30, 31), fencing studies have shown a noticeable lack of research on RAE. Fencing is an Olympic sport of individual opposition (20), where psychomotor and perceptive skills prevail (32, 33). In fencing, performance has been related mainly to perceptual, neuro-physiological characteristics, and body composition, such as the amount of lean body mass (34, 35), or power and speed (22, 33, 34, 36). This contrasts with sports like judo or wrestling, where performance relies more heavily on isometric strength (24). These reported functional and anthropometric characteristics (speed, power, and lean body mass) are normally related to age and to the maturation of athletes (37). However, the relationship between the anthropometric characteristics of athletes and fencing performance is still unclear (37, 38). To the best of our knowledge, only two studies assessed RAE in fencing. One study reported that Swiss recreational female fencers exhibited RAE, while an inverse RAE was observed in the competitive level (39). Another study about the top 20 Brazilian fencers reported a RAE for several age categories using different weapons (21).

The issue of RAE in individual sports remains a subject of ongoing debate, and this study aims to contribute to that discussion. To this end, we investigated the presence of the RAE in the World Fencing Championships events of the 2022–2023 season, across cadet, junior, and senior categories. We hypothesized that: (1) RAE would be evident in the overall sample; (2) RAE would be more prevalent in male athletes than in female athletes; and (3) RAE would be more prevalent in younger age categories and in male events.

## 2 Materials and methods

### 2.1 Study design

The aim of this study was to examine the presence of the RAE in top-level fencing competitions, specifically at the 2022–2023 World Fencing Championships. For this purpose, a cross-sectional observational design was employed, using official athlete data obtained from the International Fencing Federation database (1).

First, we assessed the presence of RAE in the total sample, as well as separately for male and female athletes. Then, RAE was investigated separately for each of the 18 fencing events (divided by sex, weapon, and age group) that took place during the 2022–2023 World Fencing Championships.

### 2.2 Data collection

All the data were collected from the International Fencing Federation website (1). The data related to the 2022–2023 season and included the cadet (U17), junior (U20), and senior athletes who participated in the World Fencing Championships. The recorded variables included the following: the participants’ date of birth, competition date, weapon, sex, age category, final result at the world championship, and number of participants in the event. Decimal age was calculated as the difference between the date of competition and the date of birth of the athlete.

In fencing, age groups start on 1 January and end on 31 December (1); therefore, to determine the distribution of birthdates by quarter, we adopted the following cutoff periods: first quarter (January to March), second quarter (April to June), third quarter (July to September), and fourth quarter (October to December).

The relative result was calculated as the ratio between an athlete's final classification in the World Fencing Championship event and the total number of participants in the corresponding event.

### 2.3 Participants

Data were collected from 2,791 participants (mean decimal age: 20.4 ± 4.4 years). The sample comprised 1,216 female athletes (mean age: 20.1 ± 5.4 years) and 1,575 male athletes (mean age: 20.6 ± 5.5 years), distributed across three age categories: 713 cadets, 1,048 juniors, and 1,030 seniors. The mean ages of cadets, juniors, and seniors were 16.2 ± 0.8 years, 18.1 ± 1.5 years, and 25.6 ± 5.7 years, respectively. There were 785 saber fencers, 1,103 épée fencers, and 903 foil fencers.

Fencers were from 119 different countries. Each country was allowed to participate with a maximum of 66 fencers across the three World Fencing Championships (cadets, junior, and seniors), distributed as follows: 18 cadets (three per weapon and sex), 24 juniors (four per weapon and sex), and 24 seniors (four per weapon and sex).

### 2.4 Statistical analyses

The Statistical Package for the Social Sciences (SPSS software version 29.0, 241, IBM SPSS, Chicago, IL, USA) was used for the statistical analysis and the significance level was set at 5%. A chi-square goodness-of-fit test was used to examine whether the distribution of athletes’ birthdates across the four birth quarters deviated from an expected uniform discrete distribution (i.e., 25% in each birth quarter). This analysis was conducted for the total sample, as well as for each sex and for each one of the 18 events. Typically, in RAE studies, the observed birthdate distribution is compared to a theoretically expected one, ideally that of the underlying population. However, in studies that analyze data from multiple countries, such as the present one, obtaining official birth statistics can be challenging. Therefore, a uniform discrete distribution is commonly used as a simplified alternative for data analysis (40). In addition, a chi-square test for homogeneity was employed to compare the distribution of birth quarters between male and female athletes.

Follow-up analysis of the chi-square goodness-of-fit test included standardized residuals, Cramér's *V* for effect size, and odds ratios (OR). When significant differences were observed in the distribution of birth quarters, the standardized residuals (*R_i_*) were used to interpret the direction of the effect: values of *R_i_* ≥ 1.96 indicated overrepresentation, while *R_i_* ≤ 1.96 indicated underrepresentation (41). The effect size was assessed using Cramér's *V*. With three degrees of freedom, the effect was considered negligible if *V* < 0.058, small if 0.058 ≤ *V* < 0.173, medium if 0.173 ≤ *V* < 0.289, and large if *V* ≥ 0.289 (42).

To estimate the likelihood of athletes being born in each birth quarter compared to a reference population, binary logistic regression was applied. For this purpose, a new dataset was created by combining the observed distribution of athletes with a hypothetical reference population, assuming a uniform discrete distribution across the four birth quarters. The dependent variable represented group membership (1 = athlete; 0 = reference population), and birth quarter was entered as the independent variable. The fourth quarter (Q4) served as the reference category, in line with standard practice in RAE research (16). Odds ratios and 95% confidence intervals (95% CI) were calculated. A relevant RAE was considered present when the confidence interval of the OR did not include the value 1. The OR comparing quarter Qi to Qj reflects how much more (or less) likely athletes born in Qi are to be represented in the sample relative to those born in Qj, in relation to the reference population, with OR >1 indicating overrepresentation and OR <1 indicating underrepresentation.

In the competitions where the RAE was detected, the Kruskal–Wallis test was applied to compare the athletes’ relative results across birth quarters. In addition, Quade's non-parametric ANCOVA test followed by multiple comparisons with Bonferroni correction was conducted to compare relative results across birth quarters while controlling for the effect of decimal age.

## 3 Results

**Figure 1:**
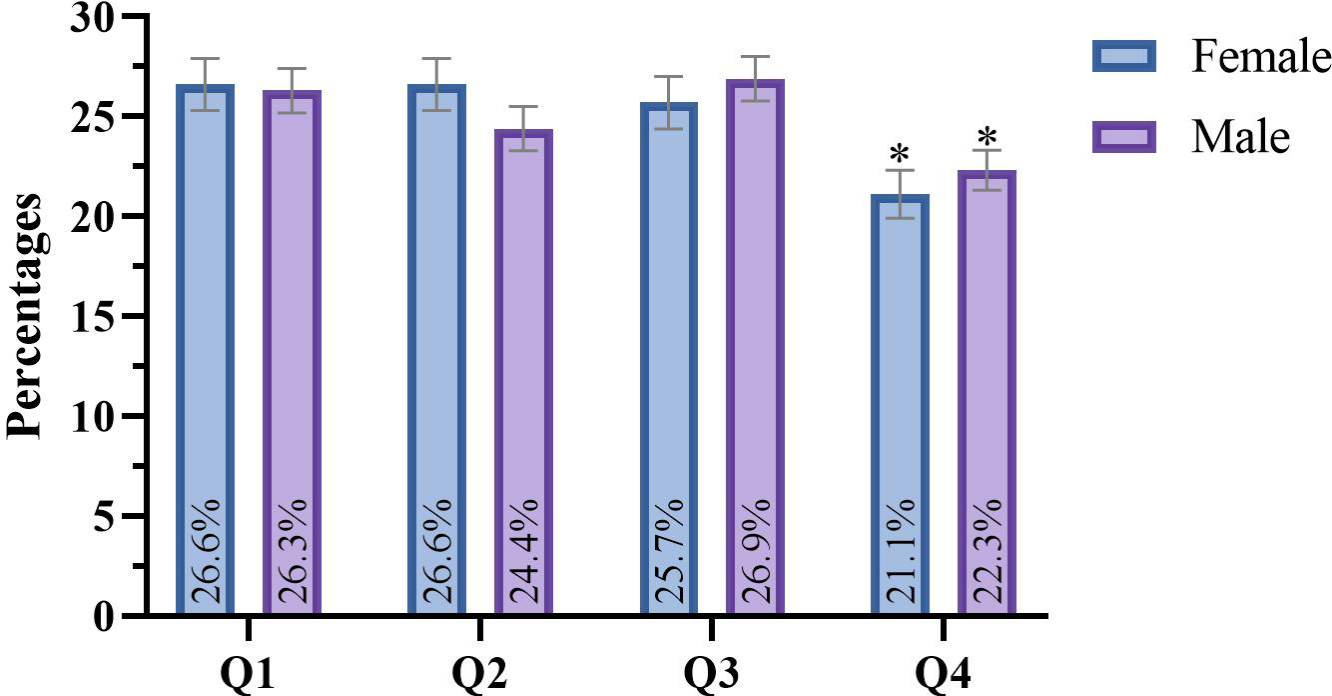
Birth quarter distribution on the overall sample of participants in the 2022–2023 World Fencing Championships. Note: Q1: first birth quarter; Q2: second birth quarter; Q3: third birth quarter; Q4: fourth birth quarter; *standardized residual ≥1.96 or ≤−1.96.

The overall sample showed significant differences in the distribution of birth quarters [*χ*^2^(3) = 16.142, *p* < 0.001], though the effect size was negligible (*V* = 0.044). The first three quarters had a higher proportion of births (Q1: 26.4% and *R_i_* = 1.49, Q2: 25.4% and *R_i_* = 0.43, Q3: 26.4% and *R_i_* = 1.49) compared to Q4 (21.8% and *R_i_* = −3.40). According to the odds ratio analysis, the likelihood of competing at the World Fencing Championships was 1.21, 1.17, and 1.21 times higher for athletes born in Q1, Q2, and Q3, respectively, compared to those born in Q4. The distribution of birth quarters is shown in Figure 1. Significant differences were also seen across birth months [*χ*^2^(11) = 38.012, *p* < 0.001, *V* = 0.034], with January showing a higher number of births (10.2% and R_i_ = 3.50), and November (7.1% and *R_i_* = −2.27) and December (6.5% and R_i_ = −3.32) showing fewer births.

**Figure 2:**
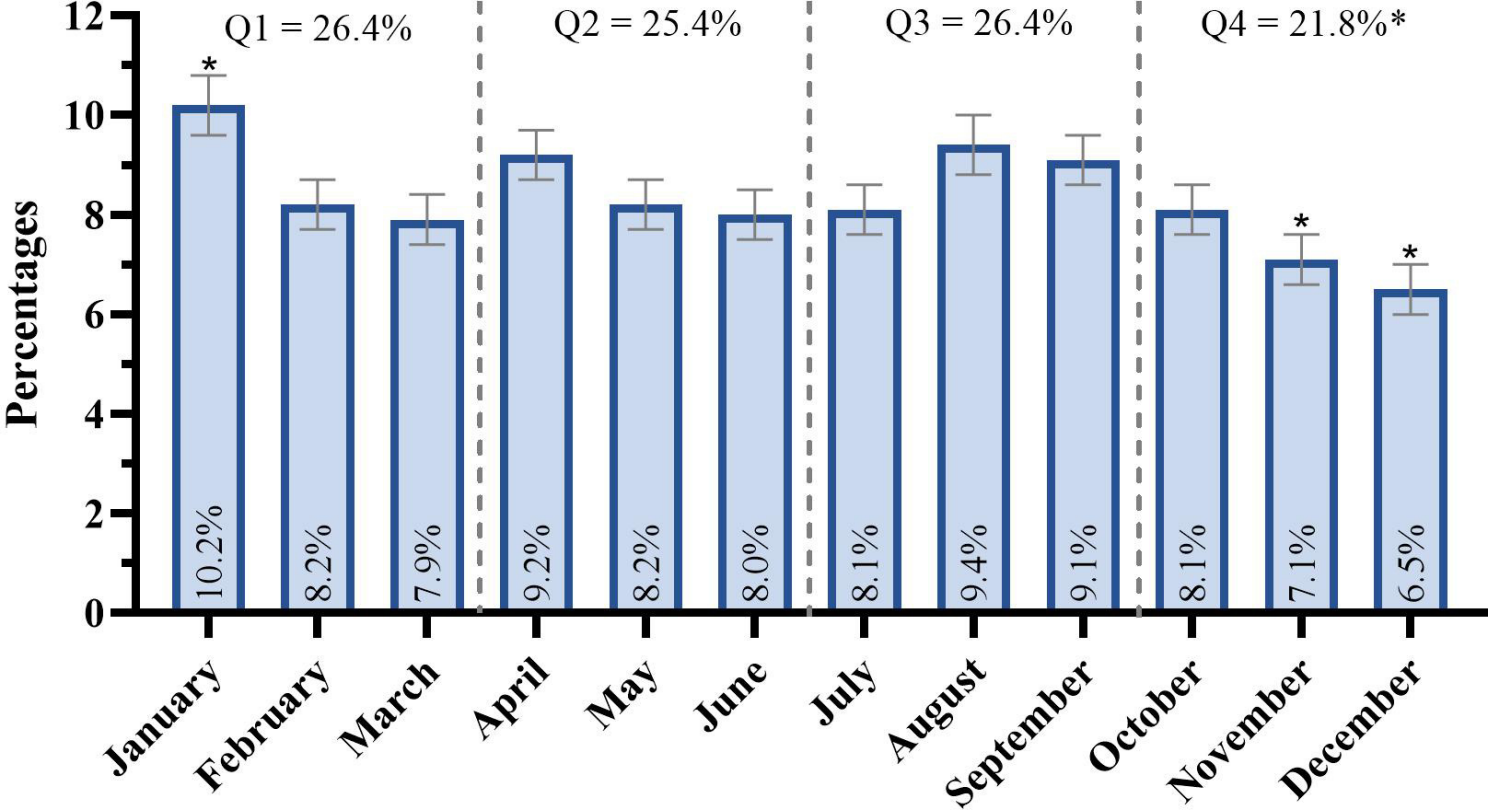
Birth quarter distribution by sex. Note: Q1: first birth quarter; Q2: second birth quarter; Q3: third birth quarter; Q4: fourth birth quarter; *standardized residual ≥1.96 or ≤−1.96.

According to sex, significant differences in the birth quarter distribution were observed for both male (*n* = 1,216) and female (*n* = 1,575) athletes (Figure 2). For female athletes [*χ*^2^(3) = 10.349, *p* = 0.016, *V* = 0.053], the distribution for Q1, Q2, Q3, and Q4 was 26.6%, 26.6%, 25.7%, and 21.1%, respectively. Athletes born in Q4 were underrepresented (*R_i_*: Q1 = 1.02, Q2 = −0.44, Q3 = 1.52, Q4 = −2.10). Based on odds ratios, the likelihood of competing at the World Fencing Championships was 1.26, 1.27, and 1.22 times higher for athletes born in Q1, Q2, and Q3, respectively, compared to Q4. For male athletes [*χ*^2^(3) = 7.987, *p* = 0.046, *V* = 0.041], 26.3%, 24.4%, 26.9%, and 22.3% of athletes were born in Q1, Q2, Q3, and Q4, respectively. There was an underrepresentation of athletes born in Q4 (*R_i_*: Q1 = 1.09, Q2 = 1.15, Q3 = 0.52, Q4 = −2.75). Based on odds ratios, the likelihood of competing at the World Fencing Championships was 1.18, 1.09, and 1.20 times higher for athletes born in Q1, Q2, and Q3, respectively, compared to those born in Q4. Furthermore, no significant differences were found in birth quarter distribution between sexes [*χ*^2^(3) = 2.219, *p* = 0.529, *V* = 0.028].

**Figure 3:**
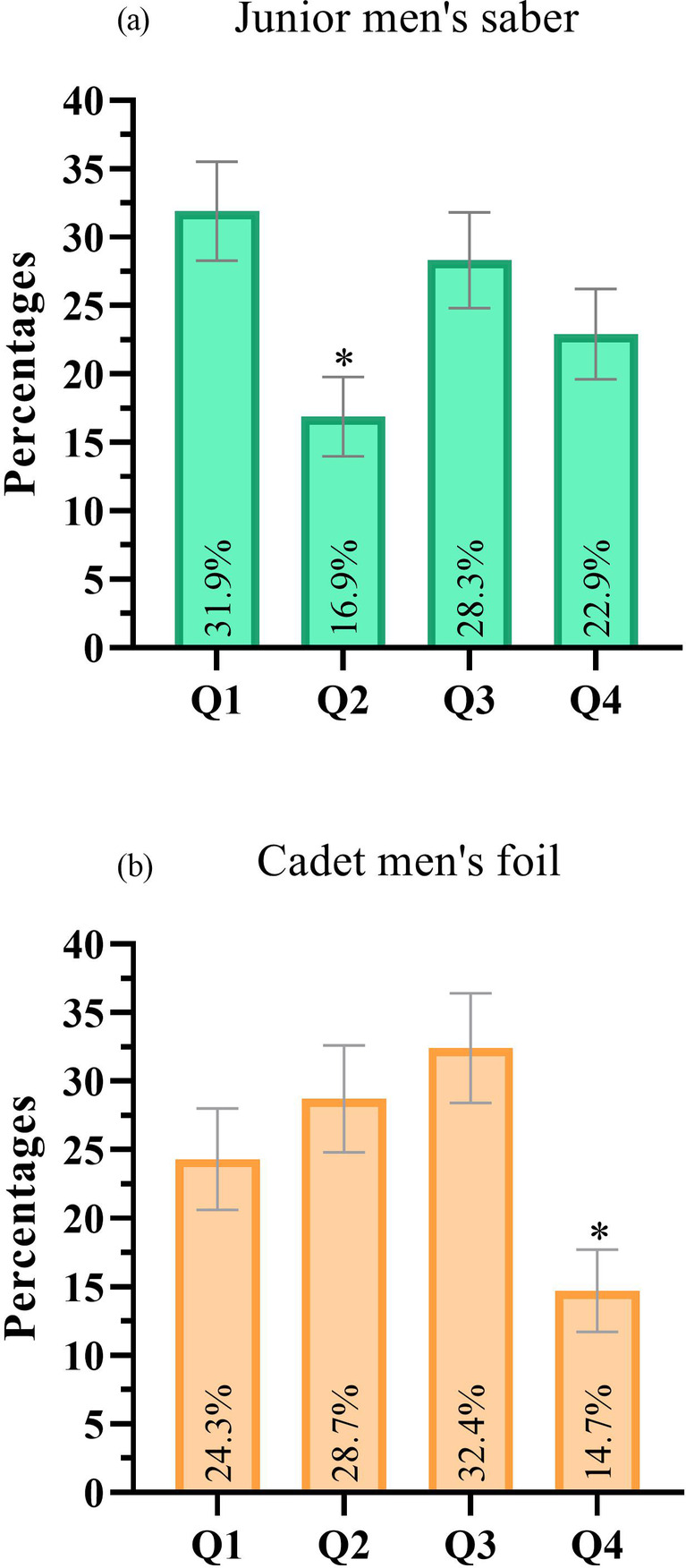
Birth quarter distribution in junior men's saber **(a)** and cadet men's foil **(b)**. Note: Q1: first birth quarter; Q2: second birth quarter; Q3: third birth quarter; Q4: fourth birth quarter; *standardized residual ≥1.96 or ≤−1.96.

Birth quarter distribution was also examined by event (Table 1). Significant differences were found in junior men's saber [*χ*^2^(3) = 8.602, *p* = 0.035, *V* = 0.131] and cadet men's foil [*χ*^2^(3) = 9.471, *p* = 0.024, *V* = 0.152). In junior men's saber, the distribution was 31.9%, 16.9%, 28.3%, and 22.9% for Q1, Q2, Q3, and Q4, respectively (Figure 3a). Athletes born in Q2 were underrepresented (*R_i_*: Q1 = 1.79, Q2 = −2.09, Q3 = 0.85, Q4 = −0.54). In the cadet men's foil event, the birth quarter distribution was 24.3%, 28.7%, 32.4%, and 14.7% for Q1, Q2, Q3, and Q4, respectively (Figure 3b). Q4 had the lowest number of births (*R_i_* = −2.40), while the remaining birth quarters were neither over- or underrepresented (*R_i_*: Q1 = −0.17, Q2 = 0.86, Q3 = 1.71). No significant differences in birth quarter distribution were observed in any of the female events or the remaining male events.

**Table 1:** Birth quarter distribution (%) of participants in the 2022–2023 World Fencing Championships and comparison between birth quarters.

Event	*N*	Birth quarters (%)				Chi-square			Odds ratio (95% CI)		
		Q1 (%)	Q2 (%)	Q3 (%)	Q4 (%)	$\$\chi^{2}\$$	*P*	*V*	OR Q1 VS. Q4 (95% CI)	OR Q2 VS. Q4 (95% CI)	OR Q3 VS. Q4 (95% CI)
Female	1,216	26.6	26.6	25.7	21.1	10.349	.016	0.053	1.26 (1.00, 1.59)	1.27 (1.01, 1.59)	1.22 (0.97, 1.54)
Saber											
Cadet	92	32.6	25.0	25.0	17.4	4.261	.241	0.124	1.88 (0.81, 4.33)	1.44 (0.61, 3.40)	1.44 (0.61, 3.40)
Junior	124	22.6	29.0	27.4	21.0	2.194	.539	0.077	1.08 (0.52, 2.24)	1.39 (0.68, 2.81)	1.31 (0.64, 2.67)
Senior	127	22.0	32.3	22.8	22.8	3.614	.314	0.097	0.97 (0.47, 1.97)	1.41 (0.71, 2.80)	1.00 (0.49, 2.04)
Epée											
Cadet	120	30.0	23.3	23.3	23.3	1.600	.668	0.067	1.29 (0.63, 2.61)	1.00 (0.48, 2.07)	1.00 (0.48, 2.07)
Junior	174	29.9	23.0	23.6	23.6	2.230	.538	0.065	1.27 (0.71, 2.28)	0.98 (0.53, 1.79)	1.00 (0.55, 1.83)
Senior	184	25.0	26.1	27.2	21.7	1.217	.748	0.047	1.15 (0.64, 2.07)	1.20 (0.67, 2.16)	1.25 (0.70, 2.24)
Foil											
Cadet	110	30.0	27.3	25.5	17.3	3.964	.266	0.110	1.74 (0.80, 3.76)	1.58 (0.72, 3.45)	1.47 (0.67, 3.24)
Junior	156	25.6	25.6	29.5	19.2	3.385	.344	0.085	1.33 (0.70, 2.55)	1.33 (0.70, 2.55)	1.53 (0.81, 2.91)
Senior	129	23.3	29.5	26.4	20.9	2.132	.555	0.074	1.11 (0.55, 2.27)	1.41 (0.70, 2.82)	1.26 (0.62, 2.54)
Male	1,575	26.3	24.4	26.9	22.3	7.987	.046	0.041	1.18 (0.96, 1.44)	1.09 (0.89, 1.34)	1.20 (0.99, 1.47)
Saber											
Cadet	106	27.4	30.2	26.4	16.0	4.261	.188	0.116	1.71 (0.76, 3.82)	1.88 (0.85, 4.18)	1.65 (0.73, 3.70)
Junior	166	31.9	16.9	28.3	22.9	8.602	.035*	0.131	1.39 (0.77, 2.54)	0.74 (0.38, 1.41)	1.24 (0.67, 2.27)
Senior	170	24.7	22.9	25.9	26.5	0.464	.926	0.030	0.93 (0.51, 1.70)	0.87 (0.47, 1.59)	0.98 (0.54, 1.77)
Epée											
Cadet	149	33.6	24.2	20.8	21.5	6.195	.103	0.118	1.56 (0.83, 2.95)	1.13 (0.58, 2.17)	0.97 (0.50, 1.90)
Junior	233	24.5	26.6	25.3	23.6	0.459	.931	0.026	1.04 (0.62, 1.74)	1.13 (0.67, 1.88)	1.07 (0.64, 1.80)
Senior	243	23.0	27.6	29.6	19.8	5.774	.124	0.089	1.17 (0.69, 1.97)	1.40 (0.84, 2.33)	1.50 (0.90, 2.50)
Foil											
Cadet	136	24.3	28.7	32.4	14.7	9.471	.024*	0.152	1.65 (0.79, 3.43)	1.95 (0.95, 4.00)	2.20 (1.08, 4.48)
Junior	195	23.1	23.1	30.3	23.6	2.887	.415	0.070	0.98 (0.55, 1.73)	0.98 (0.55, 1.73)	1.28 (0.74, 2.23)
Senior	177	27.7	20.9	22.6	28.8	3.136	.375	0.077	0.96 (0.54, 1.70)	0.73 (0.40, 1.31)	0.78 (0.44, 1.41)
Total	2,791	26.4	25.4	26.4	21.8	16.142	<0.001	0.044	1.21 (1.04, 1.41)	1.17 (1.00, 1.36)	1.21 (1.04, 1.41)

Q1: first birth quarter; Q2: second birth quarter; Q3: third birth quarter; Q4: fourth birth quarter; *V*, Cramer's *V*; OR, odds ratio; CI, confidence intervals.

*Significant difference.

When comparing the athlete's relative results across birth quarters, no significant differences were observed in the junior men's saber category (*H* = 2.389, *p* = 0.496), nor in the cadet men's foil category (*H* = 0.224, *p* = 0.974). When accounting for the decimal age effect, since older athletes are expected to be more experienced (43), the results were still not significant. In the junior men's saber category, no significant differences were observed [*F (*3, 162*)* = 1.292, *p* = 0.279], nor in the cadet men's foil category [*F (*3, 132*)* = 0.567, *p* = 0.638].

In the analysis of the overall top eight athletes per event, RAE was identified, albeit with a small effect [*χ*^2^(3) = 9.056, *p* = 0.029, *V* = 0.145]. However, the *post hoc* analysis using standardized residuals revealed no quarters outside the range of −1.96–1.96 (*R_i_*: Q1 = −1.50, Q2 = 1.67, Q3 = 1.33, Q4 = −1.50). However, when analyzing each sex and each event, no significant differences in birth quarter distributions were observed (Table 2).

**Table 2:** Birth quarter distribution (%) of the top eight fencers in the 2022–2023 World Fencing Championships and comparison between birth quarters.

Event	*N*	Birth quarters (%)				Chi-square			Odds ratio (95% CI)		
		Q1	Q2	Q3	Q4	$\$\chi^{2}\$$	*P*	*V*	Q1 VS. Q4	Q2 VS. Q4	Q3 VS. Q4
Female	72	20.8	33.3	26.4	19.4	3.444	.348	0.126	0.93 (0.35, 2.48)	0.58 (0.23, 1.48)	0.74 (0.29, 1.91)
Saber											
Cadet	8	50.0	25.0	25.0	0.0	1.000	.744	0.204	–	–	–
Junior	8	0.0	62.5	25.0	12.5	3.250	.296	0.368	–	0.20 (0.01, 3.66)	0.50 (0.02, 11.09)
Senior	8	37.5	37.5	12.5	12.5	2.000	.654	0.289	0.33 (0.02, 6.65)	0.33 (0.02, 6.65)	1.00 (0.03, 29.81)
Epée											
Cadet	8	0.0	12.5	12.5	75.0	6.250	.059	0.510	–	6.00 (0.34, 107.42)	6.00 (0.34, 107.42)
Junior	8	37.5	12.5	37.5	12.5	2.000	.654	0.289	0.33 (0.02, 6.65)	1.00 (0.34, 29.81)	0.33 (0.02, 6.65)
Senior	8	37.5	25.0	25.0	12.5	1.000	.962	0.204	0.33 (0.02, 6.65)	0.50 (0.02, 11.09)	0.50 (0.02, 11.09)
Foil											
Cadet	8	12.5	25.0	50.0	12.5	3.000	.551	0.354	1.00 (0.03, 29.81)	0.50 (0.02, 11.09)	0.25 (0.01, 4.73)
Junior	8	12.5	37.5	25.0	25.0	1.000	.962	0.204	2.00 (0.09, 44.35)	0.67 (0.05, 9.47)	1.00 (0.06, 15.99)
Senior	8	0.0	62.5	25.0	12.5	3.250	.296	0.368	–	0.20 (0.01, 3.66)	0.50 (0.02, 11.09)
Male	72	16.7	30.6	34.7	18.1	7.000	.074	0.180	1.08 (0.39, 3.01)	0.59 (0.23, 1.52)	0.52 (0.20, 1.33)
Saber											
Cadet	8	12.5	37.5	25.0	25.0	1.000	.962	0.204	2.00 (0.09, 44.35)	0.67 (0.05, 9.47)	1.00 (0.06, 15.99)
Junior	8	37.5	0.0	50.0	12.5	1.750	.552	0.270	0.33 (0.02, 6.65)	–	0.25 (0.01, 4.73)
Senior	8	12.5	12.5	37.5	37.5	2.000	.654	0.289	3.00 (0.15, 59.89)	3.00 (0.15, 59.89)	1.00 (0.08, 12.56)
Epée											
Cadet	8	25.0	25.0	25.0	25.0	0.000	1.000	0.000	1.00 (0.06, 15.99)	1.00 (0.06, 15.99)	1.00 (0.06, 15.99)
Junior	8	25.0	62.5	12.5	0.0	3.250	.296	0.368	–	–	–
Senior	8	12.5	37.5	50.0	0.0	1.750	.552	0.270	–	–	–
Foil											
Cadet	8	12.5	25.0	50.0	12.5	3.000	.551	0.354	1.00 (0.03, 29.81)	0.50 (0.02, 11.09)	0.25 (0.01, 4.73)
Junior	8	12.5	37.5	25.0	25.0	1.000	.962	0.204	2.00 (0.00, 44.35)	0.67 (0.05, 9.47)	1.00 (0.06, 15.99)
Senior	8	0.0	37.5	37.5	25.0	0.250	1.000	0.102	–	0.67 (0.05, 9.47)	0.67 (0.05, 9.47)
Total	144	18.8	31.9	30.6	18.8	9.056	.029	0.145	1.00 (0.49, 2.03)	0.587 (0.30, 1.14)	0.614 (0.32, 1.19)

Q1: first birth quarter; Q2: second birth quarter; Q3: third birth quarter; Q4: fourth birth quarter; *V*, Cramer's *V*; CI, confidence intervals. When no athletes were present in a given birth quarter, the odds ratio and its 95% CI could not be calculated.

*Significant difference.

## 4 Discussion

### 4.1 General discussion

Our study examined the presence of the RAE at the 2022–2023 World Fencing Championships. As hypothesized, RAE was evident in the overall sample, with a notable underrepresentation of fencers born in the fourth quarter. An analysis of birth months revealed that the fewest athletes were born in November and December. When analyzed by sex, both male and female participants showed fewer births in the fourth quarter. However, contrary to our expectations, no significant differences in birth quarter distribution were found between sexes. RAE was identified in only two specific events: junior men's saber and cadet men's foil. In junior men's saber, there was an underrepresentation of athletes born in the second quarter, whereas in cadet men's foil, those born in the fourth quarter were underrepresented. Notably, these patterns were not associated with final rankings in either event. When considering only the top eight fencers per event, the overall sample showed a trend toward RAE, with a higher proportion of athletes born in the second and third quarters. However, no clear evidence of RAE was found when analyzing the birth distribution of the top eight athletes in each individual event.

According to Musch and Grondin (11), the presence of RAE depends strongly on the competitiveness of the sport. A sport with increased popularity, few available playing spots, and more physically driven tends to have a greater RAE, since the most developed and athletic individuals are chosen (11, 44, 45). The remaining individuals can remain practicing at less competitive levels, change for a less popular sport, or even drop out completely from sport (16, 46). For example, in French boxing, the RAE is absent at the amateur level where there is low selection pressure (28).

Fencing is not very popular despite being an Olympic sport since the first modern Olympic Games in 1896 (1). This may explain why the RAE was absent in most World Fencing Championships’ events, with the only exceptions being the junior men's saber and cadet men's foil events. Another possible explanation is that fencing does not depend, as it might appear, on physical characteristics to obtain good results, as in football or basketball (12, 17), but on combined mental, tactical, and technical skills (9, 33, 39, 47–49). Fencing competitions involve moments of high-intensity efforts, relying heavily on anaerobic metabolism during exchanges, interspersed with longer pauses dominated by aerobic activity (33, 47, 48). During a match, fencers rely on perceptual and technical skills to succeed, whether by creating touch opportunities or responding to an opponent’s actions (33, 39). In addition, since the cadet and junior categories span 2 and 3 years, respectively, there is considerable age variability as well as morphological variability, such as height and weight. Therefore, the significant presence of RAE in only two of the 18 events analyzed, along with the underrepresentation of fencers born in the second quarter at the junior men's saber event, may be attributed to chance. Similar patterns have been observed in other technical sports, such as artistic gymnastics, rhythmic gymnastics, and figure skating, where the RAE is not observed (7, 9, 19).

The RAE observed in our overall sample is in line with the results of Joyner et al. (50), who analyzed Olympic athletes from 1896 to 1996 and reported an RAE in the Summer Olympics, in both non-ball sports and individual sports, with more births observed in the first quarter (50). We then observed the results in more detail, by sex and event, since the presence of RAE may vary across factors. These analyses revealed some inconsistencies. On one hand, RAE was significantly present among both male and female fencers, consistent with findings by Smith et al. (45), who studied elite female athletes across several sports, and by Almeida-Neto et al. (21), who focused on Brazilian fencers. However, we found no significant difference in the birth quarter distribution between sexes, as was reported in studies by Baker et al. (7) and Brustio et al. (8) in sports such as skiing, figure skating, and athletics, as well as in the meta-analyses by Smith et al. (45) and Cobley et al. (16). In addition, the absence of RAE in the senior age group aligns with the findings reported by Cobley et al. (16) and Smith et al. (45).

When comparing our findings with previous research on RAE and fencing, of which, to the best of our knowledge, there are only two studies, some inconsistencies were revealed. Romann et al. (10), who studied Swiss athletes, found no evidence of RAE among Swiss male fencers, whereas our results indicate a significant RAE in male fencers overall and specifically in two male events (junior men's saber and cadet men's foil). For female athletes, Romann et al. (10) reported the presence of RAE only at lower competitive levels and, in contrast to our findings—which identified RAE in the overall female sample—an inverse RAE at higher levels of competition. Almeida-Neto et al. (21). who examined Brazilian fencers, observed RAE in age groups younger than the cadet category. They also observed the presence of RAE among female cadets involved in the saber, epée, and foil events, as well as in the male epée category, differing from the results reported in this study. Fencing is unique among other combat sports, in that it does not employ weight classifications. Sports such as judo, wrestling, taekwondo, and boxing have often been mentioned when studying RAE in combat sports; however, to date, findings remain inconsistent. In grappling sports, such as judo and wrestling (24), Albuquerque et al. (26) reported RAE only in heavier Olympic judo categories, while Fukuda et al. (13), examining Olympic and World Championship participants, observed RAE among male athletes from cadet to senior levels across all weight classes, and RAE in female judokas competing in the heavy categories. Albuquerque et al. (24) identified RAE among male wrestlers in both Greco-Roman and freestyle disciplines. In contrast, Latyshev et al. (25) reported the absence of RAE from cadet to senior levels. In striking sports, which rely heavily on power and speed, as in fencing (24), the presence of RAE is not consistent. For example, RAE was observed in taekwondo among the top 20 Brazilian athletes (21) and at the 2018 Buenos Aires Youth Olympic Games (51); however, the absence of RAE in a sample of Olympic athletes was attributed to the sport's low popularity (27). In boxing, Kim et al. (29) reported RAE in the youth male category but not at the senior level. In contrast, RAE was evident in both male and female French boxers within the 14–15 year age categories, though it was absent at the professional level (28).

Similar to our findings, several studies on combat sports have found no association between competitive performance and the RAE (24, 25, 28, 29). This means that coaches and supporting staff should not overlook younger athletes when selecting for major competitions.

### 4.2 Practical implications and possible solutions

RAE is a consequence of grouping athletes based on chronological age. In youth sports, there can be an age difference of up to 23 months (2), as shown in the cadet age group; however, in the junior category, it can reach a difference of 35 months, since it covers a 3-year span. Consequently, at the cadet level, RAE should be more prevalent in male athletes, since most of the female athletes are already mature by the age of 16–17 years, while male athletes may still be growing (52).

Despite the inconsistency of our results and the lack of alignment with the findings of Almeida-Neto et al. (21) and Romann and Fuchslocher (39), it remains important to alert coaches to the topic of the RAE. Much of the research on RAE reports a lack of stimulation among older athletes and overstimulation at younger ages (53, 54). In fencing, training typically involves athletes of different ages and body sizes sparring with one another, which naturally counteracts the effects of the RAE. However, during competitions, athletes compete against their own age group.

One possible solution for this might be to implement a hybrid training structure, where on some days fencers are organized according to their biological age (bio-banding or estimated development age) (53, 55) or, since body dimensions might be relevant to performance, according to their height. This approach could help coaches not to overtrain athletes when they show better results, mainly due to their size, and reduce the dropout rate due to overtraining, injury, burnout, or even boredom (16).

### 4.3 Limitations and future directions

To the best of our knowledge, this is the first study focusing exclusively on RAE within the sport of fencing. Since there is very little or no previous research on this specific topic, this limitation could also serve as an opportunity to identify gaps in the literature and highlight further areas of study and development. This study, like many others, assumes an equal distribution of births across trimesters. While this approach is commonly used in research involving international samples, it may not accurately reflect real-world birth patterns, which can vary across quarters. A valuable direction for future research would be to analyze birth distributions on a country-by-country basis, allowing for more accurate assessments of the RAE in different contexts.

Our interpretation of the RAE as a causal factor may be limited by the absence of key variables related to maturation status, such as height and weight. The inclusion of these measures could have provided a more accurate understanding of the role of biological maturity on the observed patterns.

Although our primary aim was not to examine the relationship between the RAE and competitive performance, we addressed this relationship in the events where the RAE was observed. Nevertheless, we believe this topic warrants further investigation. In our findings, performance did not appear to be related to either the athlete's birth quarter or decimal age. However, other factors, such as maturation, and social, economic, political, and emotional (SEPE) influences (56), might be important.

This study highlights that RAE is less consistent in individual than in team sports, as stated by Baker et al. (7). The aim of our study was to contribute to the understanding of the RAE in fencing, as our results differ from related research. To further understand the RAE in fencing and the results presented in this study, further research is needed on the sport, examining the anthropometric characteristics and physical performance, especially strength and power, of fencers according to different weapons.

Coaches should consider the overall RAE results and provide equal opportunities to every athlete, as this can increase their chances of long-term success, as mentioned by Tascioglu et al. (17). According to Gil et al. (9) and Brustio et al. (8), younger athletes (those born later in the year) who are given the opportunity have a higher chance of a successful transition to the senior level. By taking the RAE into account, coaches can more easily ensure equal playing opportunities for all athletes, tailor training with appropriately challenging exercises for both older and younger fencers, and postpone talent selection (16, 53, 54), thereby helping to mitigate the RAE.

## 5 Conclusions

Although the RAE was present in the overall sample and when analyzed by sex, it was not consistently observed across individual World Championships events, except for junior men's saber and cadet men's foil. This suggests causality may partially explain the observed RAE, making its presence in each event uncertain. The complexity of findings in individual sports, combined with the limited research on fencing, makes it difficult to understand the significance of RAE in this sport. However, it is important to recognize that coaches can benefit from considering the relative age of athletes. Further research is needed to draw more definitive conclusions.

## Data Availability

The raw data supporting the conclusions of this article will be made available by the authors, without undue reservation.
